# Efficacy of 0.2% hyaluronic acid in the healing of skin abrasions in rats

**DOI:** 10.1016/j.heliyon.2021.e07572

**Published:** 2021-07-13

**Authors:** Marcel Nani Leite, Marco Andrey Cipriani Frade

**Affiliations:** Division of Dermatology, Department of Internal Medicine, Ribeirão Preto Medical School, University of São Paulo, Ribeirão Preto, São Paulo, Brazil

**Keywords:** Dermabrasion, Hyaluronic acid, Wound healing

## Abstract

Acute injuries, such as surgical and traumatic, heal normally in an organized and rapid manner. Studies point to the healing activity of hyaluronic acid in all phases of healing. The aim was to evaluate the effectiveness of hyaluronic acid in skin abrasions on the dorsum of rats to compare to usual products on the market. Seventy-two Wistar rats were subjected to excoriation of approximately 2.0 cm^2^ on the back by dermabrasion. According to the treatment, 3 groups were established: saline, chlorhexidine digluconate and 0.2% hyaluronic acid for 14 days. Animals were photographed on the 2^nd^, 7^th^, 10^th^ and 14^th^ postinjury days, and the index of healing of the abrasions was calculated. Biochemically, myeloperoxidase measurements of skin biopsies in addition to histological studies to assess the crust and epidermal layers were performed. The group treated with hyaluronic acid showed better re-epithelialization from the other groups (p < 0.05) on the 7^th^ and 10^th^ days. For the thickness of the crust, the hyaluronic acid group presented thinner crust than other groups on the 10^th^ and 14^th^ days (p < 0.05), but in the epidermis, no difference was observed between the groups studied. All groups showed an increase in myeloperoxidase enzyme on the 2^nd^ day, but a decreasing on the 7^th^ day. On the 10^th^ day, there was a difference in the hyaluronic acid group compared to the other groups (p < 0.05). The application of 0.2% hyaluronic acid significantly accelerated the re-epithelialization of skin abrasions compared to saline and chlorhexidine digluconate.

## 1 Introduction

Wound healing is a complex biological process in which events occur in a coordinated and sequential manner involving interactions of various cell types, matrix components, proteases and cytokines [1]. Healing by first intention occurs by minimizing the volume of connective tissue deposited, generating minimal scarring and restoring the epithelial barrier against infections [2, 3, 4]. This kind of lesions affect only the superficial layer of the skin or mucous membranes, presenting a solution of continuity of the tissue, without loss or destruction of it, with slight bleeding, but they are usually extremely painful and do not represent a risk to the victim when isolated. In general, these lesions are caused by a sharp or blunt instrument [5] and are similar to the superficial ulcers caused by the use of personal protective equipment (PPE), including face masks, continuous positive airway pressure (CPAP) masks and other devices among health professionals and patients, situations that have risen starkly during the current coronavirus disease (COVID-19) pandemic [6]. In this case, tissue repair occurs by re-epithelialization of the tissue, anatomical repair and an almost imperceptible scar. Antiseptic products are used to clean the wound or only saline is used to maintain moisture.

Hyaluronic acid (HA) was discovered in 1934 and has since been extensively studied. It has been used widely in the field of medicine, for example, in orthopedics and cosmetic surgery, but advances in treatments are in tissue repair. One of the primary functions of HA as an integral part of the extracellular matrix (ECM) is the structural role [7, 8]. Hyaluronic acid appears to play a role in all phases of wound healing, from inflammation to the remodeling process. At each phase, it has several functions in cell mediation and in events that take place in the ECM. In re-epithelialization, HA facilitates the proliferation of keratinocytes mediated by the CD44 receptor. In the remodeling process, the HA-rich matrix can reduce collagen deposition, contributing to scar reduction [9, 10, 11, 12].

Recent studies have shown good results in applying HA to wounds. Experimental research with animals [13, 14] and clinical studies using HA in venous ulcers showed decreased wound closure time and scar improvement [15].

Studies in the literature showing the effectiveness of HA in excoriations are scarce, even being a simpler lesion with easy resolution. However, which the increased use of protective masks during the COVID-19 Pandemic, for example, its frequency became higher. Thus, considering the diversity and the increasing of the abrasions in the current population and the consequent clinical-functional, social and financial disorders, it is relevant to investigate the efficiency of treatment with topical hyaluronic acid in the healing process of abrasions in an experimental model of cutaneous excoriation in rats.

## 2 Material and methods

### 2.1 Products

The products used included 0.2% hyaluronic acid cream (TRB Pharma, Campinas – SP, Brazil), antiseptic solution of chlorhexidine digluconate 10 mg/mL (Cosmed Indústria de Cosméticos e Medicamentos S.A., Barueri – SP, Brazil) and 0.9% sodium chloride sterile solution in water. All were purchased from a local pharmacy (São Paulo Pharmacy - Ribeirão Preto/São Paulo).

### 2.2 Ethics approval

The experimental protocols and handling were approved and conducted according to the ethics principles in animal research by the Ethics Commission in Animal Research (CETEA) – Ribeirão Preto Medical School, University of São Paulo, registry number 137/2016. The study was carried out following the guidelines adopted by the Brazilian College of Animal Experimentation (COBEA).

### 2.3 Animals

A total of 72 male Wistar rats weighing 180–200 g from the Central Animal Facility of the Ribeirão Preto Medical School, São Paulo, Brazil, were used. The animals were housed for 3–5 days in the vivarium for acclimatization before the surgical process, kept in individual cages with water and food ad libitum and alternating cycles of light every 12 h.

### 2.4 Surgical procedure and groups

The rats were anesthetized intraperitoneally with ketamine (70 mg/kg) and xylazine (12 mg/kg), trichotomized, and hair removal with depilatory cream was performed on the animals' backs with excess cream removed after 5 min. Subsequently, the LB-100 dermabrasion device (BELTEC Indústria e Comércio de Equipamentos Odontológicos - Araraquara, SP) was used with a diamond sandpaper RH14633 (RHOSSE Instrumentos e Equipamentos Cirúrgicos - Ribeirão Preto-SP), where it was passed on the animal's back to achieve a skin excoriation of approximately 2.0 cm^2^. After surgery, 50 mg/kg of dipyrone diluted in saline was administered intraperitoneally 2 times a day (12/12 h) for the first 24/48 h, depending on the animals' behavioral changes regarding pain. The animals were randomly divided into three groups, with 24 rats each and their injured areas were treated topically with (sufficient amount to cover the lesion), once a day, for 14 days with 0.2% hyaluronic acid cream (HA), 10 mg/mL chlorhexidine digluconate as a positive control (CD) or 0.9% sodium chloride solution (saline) (S). No occlusive dressing was used [16].

### 2.5 Study of the re-epithelization of abrasions

After the surgical process, images were taken using the basic mode of a Sony DSC-P100 digital camera of the dorsal lesions of each animal, representing day 0 (initial day), using a metallic apparatus where the camera was fixed on an aluminum base with a millimeter ruler at a standard distance of 30 cm and perpendicular to the lesion. The abrasions were photographed on days 2, 7, 10 and 14 and subsequently analyzed by ImageJ software to calculate the abrasion healing rate (AHR), calculated by the formula [(initial area - final area)/initial area] as described and performed by Leite et al. [16].

### 2.6 Harvesting material for study

Six animals from each group were euthanized on the 2^nd^, 7^th^, 10^th^ and 14^th^ days using an overdose of anesthetic, and samples were collected for further studies. The animals had all their skin cut around the abrasions using scissors, and a cylindrical fragment from the region of the lesion was collected, which was stored in a 10% buffered formalin solution for histological studies (hematoxylin-eosin) and another in a freezer at -80 °C for biochemical measurement of the myeloperoxidase enzyme (MPO) to determine the neutrophilic infiltrate.

### 2.7 Histological study–measurement of the crust and epidermis

The biopsies were kept packed for 24 h in a 10% buffered formalin solution and embedded in paraffin following the protocol of Andrade et al. [17]. For capture and analysis of histological images, a Leica DM 4000B® optical microscope equipped with a LEICA DFC® 280 camera (Leica Microsystems, Germany) was used. The images were calibrated from 242 pixels to 200 μm using the Leica Application Suite (LAS) software version 3.2.0. Subsequently, a line was drawn from the granular layer of the epidermis until the transition with the dermis, to measure the epidermis thickness and crust thickness. At the end of the tracing, the software provided the distance in micrometers [16].

### 2.8 Myeloperoxidase (MPO) measurement

Skin segments were cut into small pieces, weighed and immersed in 300 μl of a buffer solution containing 0.1 M sodium chloride (NaCl) plus 15 mM 2,2′,2'',2'''-(Ethane-1,2-diyldinitrilo) tetraacetic acid (EDTA) diluted in 20 mM sodium phosphate (NaPO_4_) for homogenization. Then, the tissue was centrifuged at 600 x g for 15 min at 4 °C, and the supernatant was discarded. The pellet was resuspended by vortexing in 1 mL of a cold 0.2% NaCl solution to lyse the red blood cells. The pellet obtained after centrifugation was resuspended in 300 μl of a buffer solution containing 0.5% hexadecyl-trimethyl ammonium bromide (H-TAB) diluted in 50 mM NaPO_4_ (Sigma-Aldrich Chemical Co., St. Louis, MO). To finish the protein extraction, the sample was frozen and thawed 3 times and centrifuged at 12,000 x g for 15 min at 4 °C. The supernatant was used for the enzymatic assay, which was performed in 96-well plates containing 25 μl of sample and revealed by the addition of 3.3′, 5.5′-tetramethylbenzidine (TMB). The reaction was interrupted by the addition of 4 M H_2_SO_4_, and the reading was performed on a spectrophotometer at 450 nm.

### 2.9 Statistical analysis

Data are expressed as the mean value ±SEM (standard error of the means). For all methods, the statistical test of analysis of variance was applied for multiple comparisons (one-way ANOVA and Tukey posttest). GraphPad Prism 6.0 software was used to make the graphs and perform statistical tests. Values of p < 0.05 show statistical evidence that there is a difference between the data in question under a 95% confidence interval.

## 3 Results

### 3.1 Evolution of the re-epithelialization process

**Figure 1:**
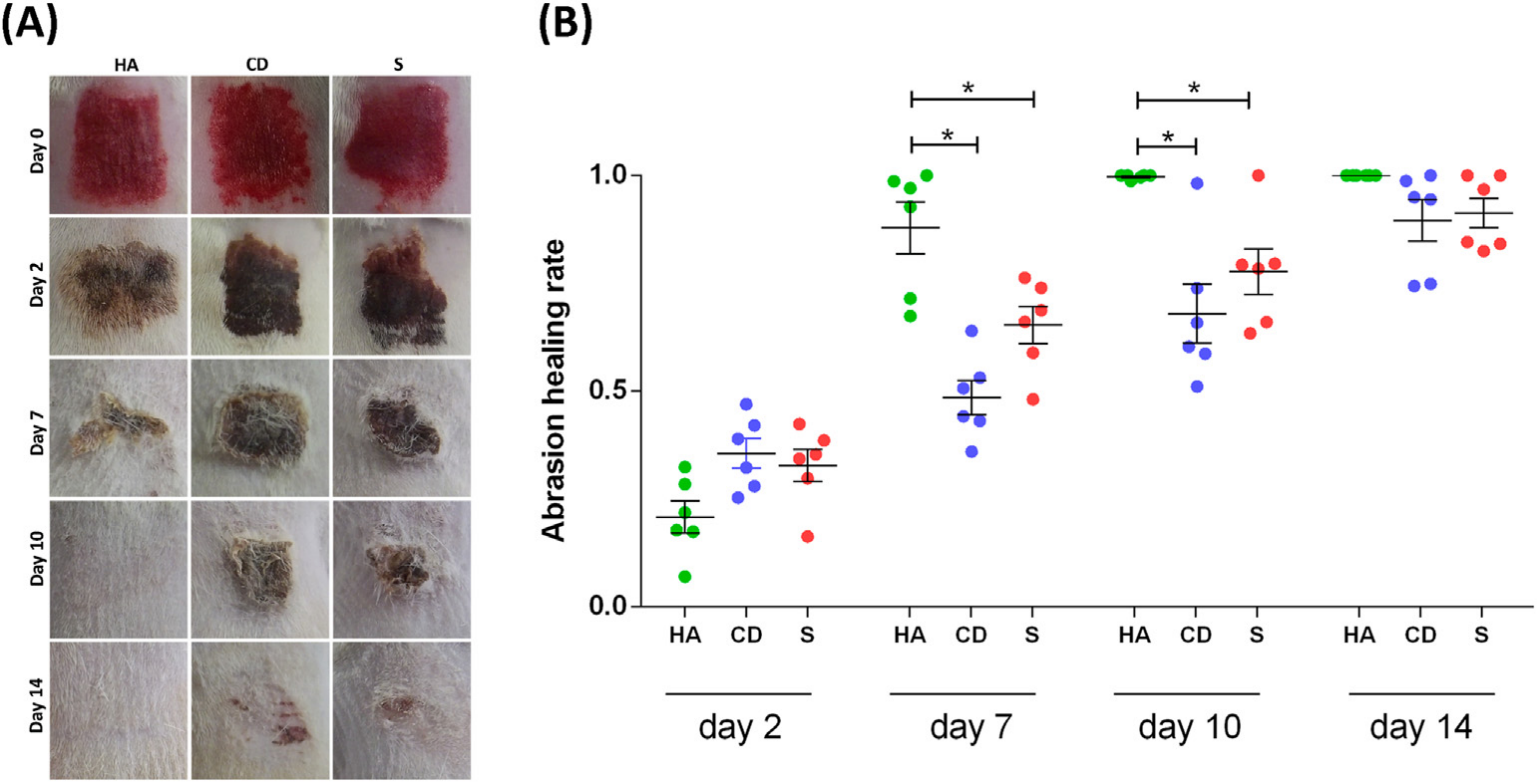
Evaluation of treated exulceration. (A) Clinical follow-up of cutaneous abrasions. (B) Quantification of re-epithelialization treated with HA, CD and S on days 2, 7, 10 and 14. ANOVA statistical test, Tukey posttest. ∗Corresponds to a significant difference (p < 0.05).

The evolution of the re-epithelialization process was assessed by the AHR. On the 2^nd^ day of follow-up, there was no evidence of a difference between the three groups studied. On days 7 and 14, the HA group presented re-epithelialization rates that were higher than the other groups (p < 0.05), with practically all lesions re-epithelialized. On the 14^th^ day, even without a difference, the excoriations of the HA group were all re-epithelialized differently from the other groups that still had a crust and a delay in re-epithelialization of more than 50% (Figure 1A, B).

### 3.2 Histopathological evaluation

**Figure 2:**
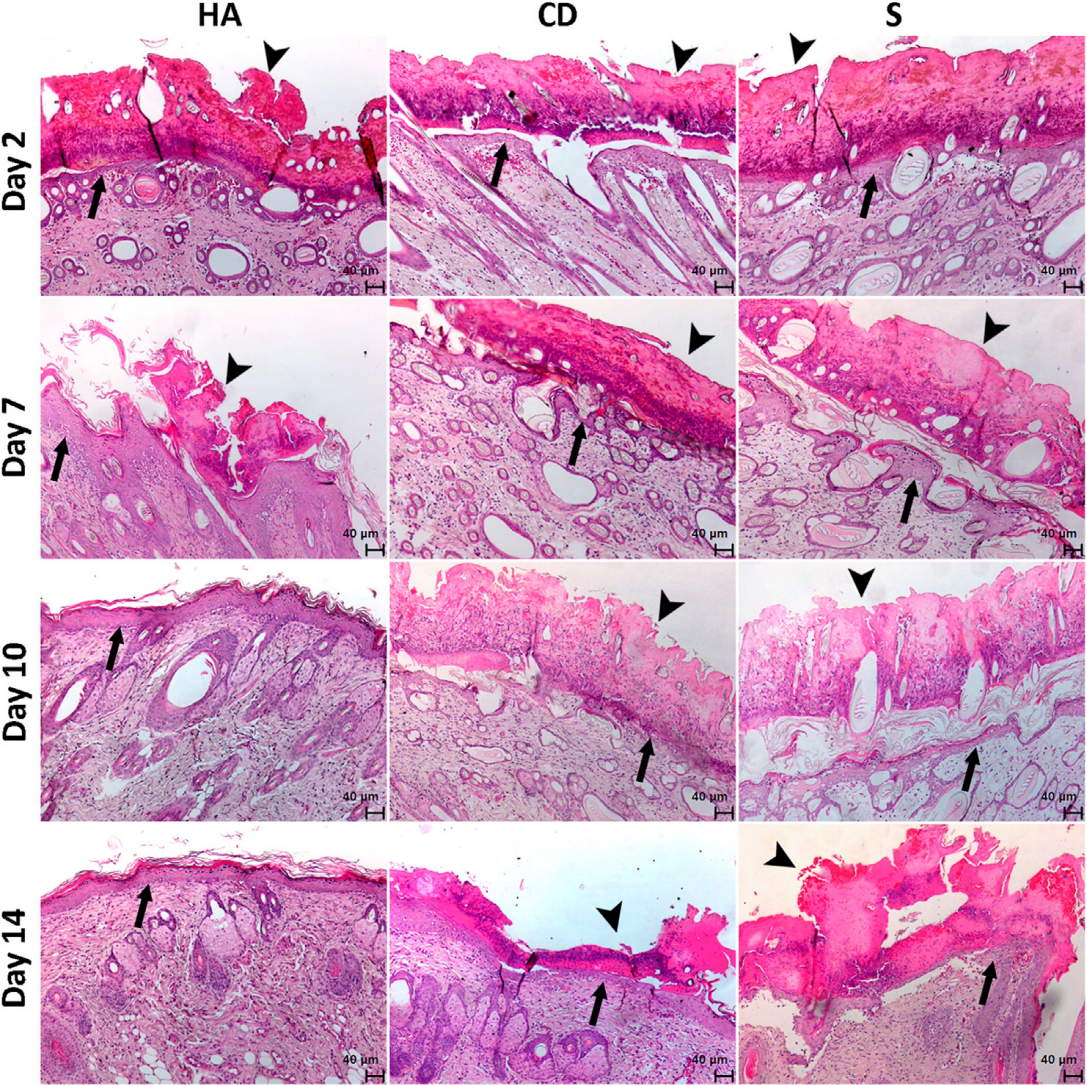
Photomicrography of the exulcerated areas. Photomicrography of the excoriated areas treated topically with HA, CD and S at 2, 7, 10 and 14 days of follow-up; samples were stained with hematoxylin-eosin (HE). The black arrows indicate the thickness of the epidermis, and the black arrow heads indicate the thickness of the crust (magnification: 50x).

**Figure 3:**
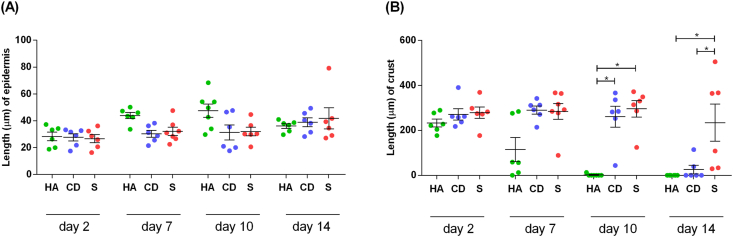
Average thickness of the crust and epidermis of the exulcerated areas. (A) Distribution of the mean thickness (μm) of the epidermis and (B) distribution of the mean thickness (μm) of the crust of the groups treated with HA, CD and S on days 2, 7, 10 and 14. Values represent the means ± SEM. ANOVA statistical test, Tukey posttest. ∗Corresponds to a significant difference (p < 0.05).

Through hematoxylin and eosin staining, histological analysis of posttreatment lesions was performed on follow-up days 2, 7, 10 and 14. Regarding epidermal thickness, although the groups showed similar results, there was a tendency toward an increase in the number of keratinocyte layers in the group treated with HA on days 7 and 10, resulting in a more consistent and resistant re-epithelialization (Figures 2 and 3A).

Regarding the thickness of the crust, on the 2^nd^ day, there was no difference between treatments. Although there is no evidence of a difference on the 7^th^ day, a large decrease in crust can be observed in the HA group in more than 50% of the treated animals. On day 10, practically all animals in the HA group did not have a crust and were re-epithelialized, corroborating with the AHR (Figure 1A-B) and different from the CD and S groups (p < 0.05) in which important evidence was still observed of crusts, a factor involved in the delay of re-epithelialization. On the 14^th^ day, the S group still contained a different crust than the HA groups, which had no more crust and were already re-epithelialized, and the CD group in which a decrease in the crust could be observed (p < 0.05) (Figures 2 and 3B).

### 3.3 Biochemical evaluation: myeloperoxidase (MPO) measurement

**Figure 4:**
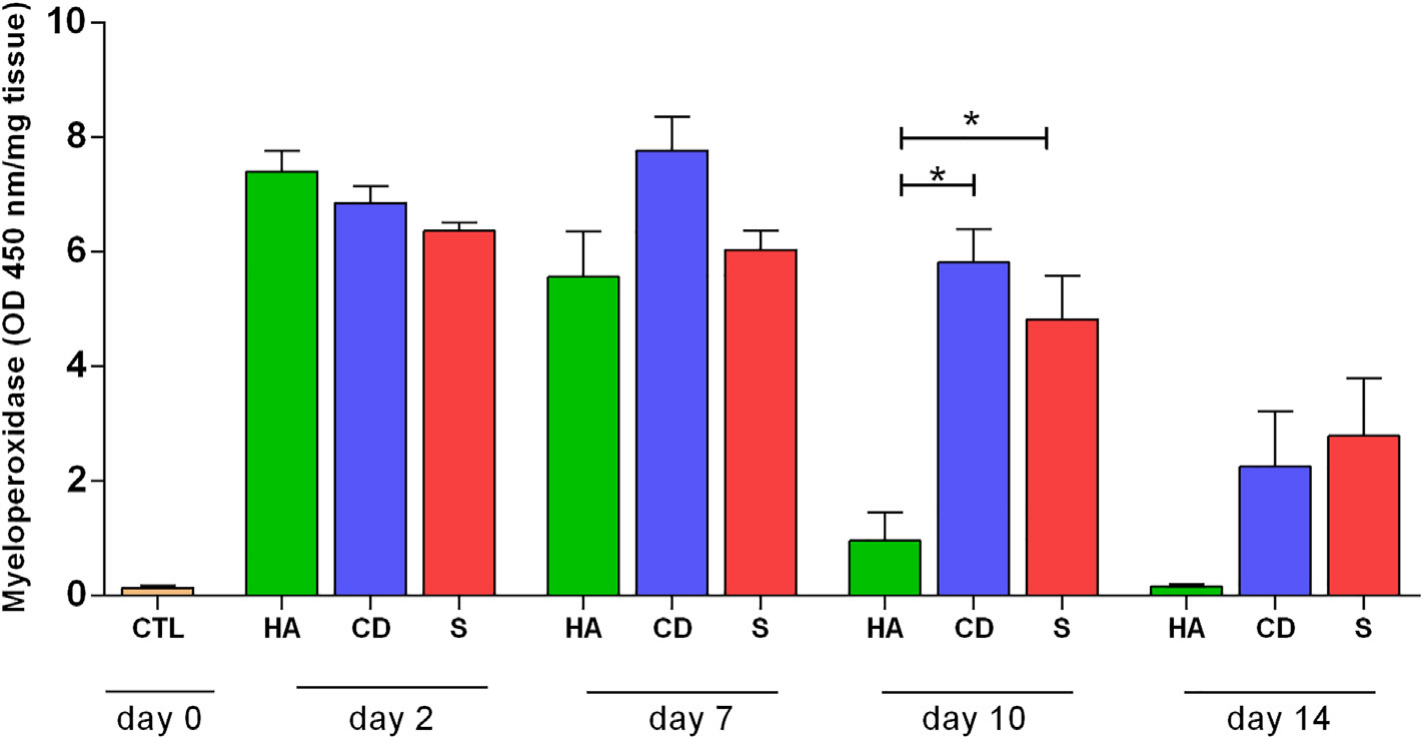
MPO levels on exulcerations treated. Quantification of the myeloperoxidase enzyme (MPO) of cutaneous abrasions treated topically with HA, CD and S on days 2, 7, 10 and 14. Values represent the means ± SEM. ANOVA statistical test, Tukey posttest. ∗Corresponds to a significant difference (p < 0.05).

To evaluate the inflammatory process with the products used in the study, a colorimetric enzymatic assay was performed by indirect measurement of neutrophils. On the 2^nd^ day, an increase in the MPO enzyme level was observed in all groups studied. On the 7^th^ day, a decrease in the enzyme was observed in the HA group, despite having no evidence of a difference. On the 10^th^ day of treatment, there was a large decrease in the enzyme in the HA group compared to the other groups (p < 0.05). Even without evidence of a difference on the 14^th^ day, the HA group showed similarity to the control (CTL) (without treatment – initial day) (Figure 4).

## 4 Discussion

The most important treatment for wounds is closure with recovery of functions and morphology. First intention wounds, including abrasions, usually occur with domestic accidents and aesthetic procedures that involve abrasion. For these wounds, treatments are carried out to reduce pain and to keep the wound moist [16, 18, 19, 20].

There are few studies in the literature on excoriation in animals and their treatment. Our work shows the effectiveness of 0.2% hyaluronic acid cream compared to a product used for this type of lesion, namely, chlorhexidine digluconate (antiseptic), and saline used to keep the lesion moist.

HA has important functions in the healing process. One of them is to increase the expression of CD44, a transmembrane glycoprotein, the main receptor for HA and presented in many cells of the human body [21]. Regarding the re-epithelialization of the excoriations, by the 7^th^ day, the animals that received HA already presented a faster re-epithelialization as compared to the other groups (p < 0.05), which occurred on the 10^th^ day. On the 14^th^ day, all animals treated with HA were already re-epithelialized, unlike the other groups that showed a clinical delay and still had a crust (Figure 1A-B). In our study, an increase in CD44 may have occurred and may be related to the fast reepithelization of excoriations in the HA group. Recent studies have used HA in a cell culture model for the proliferation of keratinocytes, which have become important in new clinical treatments for wound healing [5, 22]. Turley and Torrance [23] found that when HA is biodegraded, it produces byproducts that help with keratinocyte proliferation and migration efficiency. In addition, Leite et al. [16] used the same animal model to treat excoriations with latex and showed similar results regarding re-epithelialization and the delay when using antiseptic and saline.

The amount of crust is closely related to the inflammatory phase of healing. In this study, we observed that the animals treated with HA from the 7^th^ day onwards practically had no crust, unlike the other groups that could still be observed to have a large amount of crust. Gupta and Dai [24] used an excoriation model with BALB/c mice and showed that the animals in the untreated group still had a crust on the 8^th^ day of follow-up, similar to the results of the current study, only different from the HA group. Leite et al. [16] showed similar results in the same model regarding the amount of crust; on the 10^th^ day, only the group treated with latex had no more crust. Considering the enormous frequency of superficial skin lesions, as well as the important current impact of injuries due to individual protection tools for professionals and to therapeutic equipment for patients related to COVID-19 [6], products that promote a faster reduction in crusts, less inflammation and faster skin healing are becoming very useful and safe alternatives.

Furthermore, corroborating our results, which showed a tendency for a greater number of keratinocyte layers in the HA group in relation to the other groups and reaffirming the action of HA in the proliferation of keratinocytes, Leite et al., and Gupta and Dai [16, 24] showed fewer keratinocyte layers in the antiseptic and saline groups and in the untreated group, respectively.

The initial inflammatory phase is rich in hyaluronic acid. It promotes an increase in proinflammatory cytokines (TNFα, IL-1β and IL-8) mediated by CD44 (the largest hyaluronic acid receptor) [25, 26]. Even in the inflammatory phase, HA has a contradictory function; it can also moderate the inflammatory response by inhibiting inflammatory proteinases, thus contributing to better stabilization of granulation tissue [27]. This may be due to some fragments of HA such as high molecular weight HA (HMW HA), which has immunosuppressive anti-inflammatory properties, while on the other hand, another low molecular weight HA (LMW HA), molecule has pro-inflammatory properties [28, 29]. Thus, in our work about HA group, we suggest that a high concentration of the LMW HA molecule in the initial days (2 and 7) may have been produced because we observed an increase in the MPO enzyme. On the other hand, an increase of the HMW HA molecule on the 10^th^ and 14^th^ since may have occurred considering a decrease of inflammation status in these days, measured by MPO, lower than the other groups with a still persistent inflammation despite a decrease in MPO.

### 4.1 Limitations

In this study we used a cream with hyaluronic acid, but it was not possible to use only the cream vehicle without hyaluronic acid. In addition, the model does not allow for lesions of the same diameter, as it is made using dermabrasion device with a diamond sandpaper, thus leaving the lesion irregular. Another limitation of the study was the non-blinding of the evaluators regarding the treatment groups.

## 5 Conclusion

In view of the results and the vast literature on the positive effects of HA, the 0.2% HA cream proved to be effective in the re-epithelialization of rats submitted to excoriation lesion, when compared with antiseptic and saline solution, and can be established as an important therapeutic alternative for superficial skin lesions also in humans.

## Declarations

### Author contribution statement

Marcel Nani Leite: Conceived and designed the experiments; Performed the experiments; Analyzed and interpreted the data; Wrote the paper.

Marco Andrey Cipriani Frade: Conceived and designed the experiments; Performed the experiments; Analyzed and interpreted the data; Contributed reagents, materials, analysis tools or data; Wrote the paper.

### Funding statement

This work was supported by 10.13039/501100003593Conselho Nacional de Desenvolvimento Científico e Tecnológico (CNPq) [grant number #423635/2018-2], 10.13039/501100002322Coordenação de Aperfeiçoamento de Pessoal de Nível Superior (CAPES) [grant number #88882.180013/2018-01] and 10.13039/501100008353Fundação de Apoio ao Ensino, Pesquisa e Assistência do Hospital das Clínicas da Faculdade de Medicina de Ribeirão Preto-USP (FAEPA) [grant number 120/2020].

### Data availability statement

Data included in article/supplementary material/referenced in article.

### Declaration of interests statement

The authors declare no conflict of interest.

### Additional information

No additional information is available for this paper.
